# The procoagulant effects of fine particulate matter *in vivo*

**DOI:** 10.1186/1743-8977-8-12

**Published:** 2011-03-15

**Authors:** Evren Kilinç, Holger Schulz, Gerhardus JAJM Kuiper, Henri MH Spronk, Hugo ten Cate, Swapna Upadhyay, Koustav Ganguly, Tobias Stoeger, Manuela Semmler-Bhenke, Shinji Takenaka, Wolfgang G Kreyling, Mike Pitz, Peter Reitmeir, Annette Peters, Oliver Eickelberg, H Erich Wichmann

**Affiliations:** 1 Department of Internal Medicine, Laboratory for Clinical Thrombosis and Haemostasis, Cardiovascular Research Institute Maastricht, Maastricht University Medical Center, P.O. Box 616, UNS 50: Box 8, 6200 MD, Maastricht, The Netherlands; 2 Institute of Epidemiology I, Helmholtz Zentrum München, German Research Center for Environmental Health, Ingolstaedter Landstrasse 1, D85764, Neuherberg/Munich, Germany; 3 Institute of Epidemiology II, Helmholtz Zentrum München, German Research Center for Environmental Health, Ingolstaedter Landstrasse 1, D85764, Neuherberg/Munich, Germany; 4 Comprehensive Pneumology Center, Institute of Lung Biology and Disease, Helmholtz Zentrum München, German Research Center for Environmental Health, Ingolstaedter Landstrasse 1, D85764, Neuherberg/Munich, Germany; 5 Institute of Health Economics and Health Care Management, Helmholtz Zentrum München, German Research Center for Environmental Health, Ingolstaedter Landstrasse 1, D85764, Neuherberg/Munich, Germany; 6 Focus Network Nanoparticles and Health, Helmholtz Zentrum München, German Research Center for Environmental Health, Ingolstaedter Landstrasse 1, D85764, Neuherberg/Munich, Germany; 7 Department of Environmental and Occupational Health, Graduate School of Public Health, University of Pittsburgh, PA, Bridge Side Point; 100 Technology Drive, Pittsburgh, 15219 PA, USA

## Abstract

Inhalation of fine particulate matter (<2.5 μm; fine PM) has been shown to increase the risk for cardiovascular events. In this letter, we reappraise the role of tissue factor (TF) antigen and we also summarize changes in measured coagulation proteins in humans and rodents by other studies with fine PM. By considering all studies including ours, we conclude that monitoring the overall coagulation state by measuring capacity assays such as thrombin generation, and quantification of TF activity would be more suitable than determining single coagulation proteins (such as TF antigen) in order to better assess the systemic prothrombotic effects of fine PM.

## Blood coagulation markers and fine PM exposure studies

Evren Kilinç, Gerhardus JAJM Kuiper, Henri MH Spronk, Hugo ten Cate

Earlier epidemiologic and experimental studies have shown that fine particulate matter (<2.5 μm; fine PM) inhalation is associated with arterial and venous thrombosis, as well as an increased risk for cardiovascular death [1]. Several studies have addressed the possible mechanisms involved in PM-related arterial and venous thrombosis, although the recently published work by Emmerechts et al. could not establish a direct effect of intratracheal PM instillation on induction of venous thrombosis in mice [2]. Tissue factor (TF) is expressed in subendothelial cells upon injury, or on the surface of cells like monocytes, macrophages and neutrophils. A small amount of TF is necessary to form a complex with factor VIIa to produce thrombin via the extrinsic pathway of coagulation [3]. Since PM is also related to endothelial damage and activation of platelets and macrophages upon short term in vivo exposure [1], the measurement of TF in blood may be considered as a marker of cell damage.

A recent publication in this journal, addressed the effects of ambient fine PM in spontaneously hypertensive rats (SHRs) following intratracheal instillation at varying concentrations. Surprisingly, an early *decrease *in lung specific tissue factor (TF) antigen was observed at 1 and 3 days post exposure, whereas plasminogen activator inhibitor-1 (PAI-1) was increased at 1 day post instillation of fine PM [4].

It is known that a small proportion of TF exerts prothrombotic effects and the inactive form of TF may not reflect TF activity [3]. Additionally, tissue factor pathway inhibitor (TFPI), the physiological inhibitor of TF, regulates TF activity. Therefore, the measurements of TF antigen in tissues do not necessarily reflect the functional capacity and integrity of TF. Overall, measuring TF activity could be a better approach for determining procoagulant activity in tissues (*local effect*) and plasma (*systemic effect*).

Indeed, we recently showed that short-term inhalation of fine PM *increased *lung specific TF activity at 4 and 48 hours post instillation as well as the overall procoagulant potential of lung tissue, as assessed by thrombin generation, most likely through attenuated expression and activity of the natural anticoagulant thrombomodulin [5].

In addition to rodent studies [6], different single coagulation proteins have also been measured in humans exposed to fine PM [7-11]. The outcome of such studies is rather inconsistent however, either with changes, or no change in the levels of coagulation proteins, such as factor VII (FVII), FVIII, FIX, FX. The specific responses being most likely related to differences in fine PM source and the dose used, as well as the time course of sample collection and the experimental setup. At the same time, it is likely that possible procoagulant reactions to acute PM exposure will be downplayed by an increased anticoagulant response as a systemic protective mechanism. Therefore, the balance in pro- and anticoagulant reactions to PM will modulate the net thrombotic events via thrombin, which is the critical enzyme regulating the final common pathway leading to fibrin formation [5].

In further studies of prothrombotic mechanisms of PM, either after acute or chronic exposure, we would advocate the use of overall capacity assays such as for thrombin generation, in conjunction with TF activity and specific anticoagulant proteins in order to better understand the net contribution of fine PM to thrombotic and cardiovascular events.

## Response to Kilinç et al

Holger Schulz, Swapna Upadhyay, Koustav Ganguly, Tobias Stoeger, Manuela Semmler-Bhenke, Shinji Takenaka, Wolfgang G Kreyling, Mike Pitz, Peter Reitmeir, Annette Peters, Oliver Eickelberg, and H Erich Wichmann

We appreciate the comments from Kilinç and coworkers concerning application and interpretation of blood coagulation markers in PM exposure studies. They suggest the application of capacity assays rather than measurements of single coagulation factors to better understand alterations of the coagulation homeostasis in PM exposure studies. With respect to our study [4], the functional activity of TF in target organs would have been more suitable than measurement of the antigen level in tissues. We agree with Kilinç et al. that TF activity or even further coagulation assays would certainly improve our understanding of the complex response observed in our PM2.5 Augsburg (PM2.5-AB) exposure study. Reliable activity assays evaluated in correspondence to their protein/transcription levels always provide essential functional information of biological responses. However, our study was designed to get a global impression of PM2.5-AB associated inflammatory and cardiovascular effects in target organs and their potential interactions rather than being specifically focused on disturbances of the coagulation cascade. Related to thrombogenic effects reported after PM exposures [6,10,12-16], we selected TF and PAI-1 as representative markers to assess deteriorations of the coagulation homeostasis. Increased levels of TF and PAI-1 were observed in the heart three days after exposure to high PM2.5-AB. This is in line with the common understanding that inflammatory activity - as evidenced in our study by increased levels of osteopontin and macrophage inflammatory protein (MIP)2 in the heart and C- reactive protein (CRP) in the serum - triggers TF mediated coagulation [17]. The scenario observed in the lung appears to be more complex, an early inflammatory response at day 1 is associated with a reduced TF and an increased PAI-1 level while at day 3 both markers, in particular PAI-1, were substantially down regulated. Most inflammatory markers reached baseline levels at this time point [4]. Our data indicate that PM2.5-AB exposure alters the coagulation homeostasis in lungs and heart and shows that main target organs exhibit a different response with respect to time course and direction. As already mentioned by Kilinç et al. these results are principally in line with reports from other studies whereby differences in the PM source, the route of administration, and the dose may explain the different outcomes described so far with respect to the endpoints selected. Indeed, uncovering the underlying pathomechanisms of PM2.5-AB associated effects on the homeostasis of blood coagulation warrants further investigation and would require the assessment of platelet function, major pro- and anticoagulant pathways and may be even the bidirectional interaction between inflammation and coagulation [17]. It remains a task to specifically address this issue by assessing protein levels and functional activity for a set of key coagulation events including the assessment of the tissue factor thrombomodulin balance and fibrin formation as suggested by Kilinç et al.

## Competing interests

The authors declare that they have no competing interests.

## Authors' contributions

EK, HS, GJAJMK, HMHS, HtC, SU, KG, TS, MSB, ST, WGK, MP, PR, AP, OE, HEW all equally contributed to writing this manuscript and approved the final version.
